# Supplementary material for: Analytical Nuclear Gradients for the Multiconfigurational Self-Consistent Field Method Coupled with the Polarizable Fluctuating Charges Model
Source: J Chem Theory Comput. 2026 Jan 27;22(3):1350–62. doi: 10.1021/acs.jctc.5c01890 (PMC12895414; doi:10.1021/acs.jctc.5c01890)
Supplement: Supplementary file 1 [file ct5c01890_si_001.pdf]

Supporting Information for: Analytical Nuclear Gradients for the Multiconfigurational Self-Consistent Field Method Coupled with the Polarizable Fluctuating Charges Model

Francesco Mazza, Marco Trinari, Chiara Sepali, and Chiara Cappelli*

Scuola Normale Superiore, Piazza dei Cavalieri 7, I-56126 Pisa, Italy

E-mail: chiara.cappelli@sns.it

S1 Nuclear gradients with respect to MM coordinates

The derivative of the energy with respect to the position of MM atoms (ξ_{MM}) arises from $E^{QM/MM}$ and E^{MM} energy contributions (see Equation 1 in the main text), because E^{QM} does not depend directly on MM coordinates. Using the chain rule and the variational conditions, the nuclear gradient can be expressed as:

$$\frac{dE}{d\xi_{MM}} = \frac{dE^{QM/MM}}{d\xi_{MM}} + \frac{dE^{MM}}{d\xi_{MM}} = \sum_{i\alpha} q_{i\alpha} \frac{dV_{i\alpha}(D)}{d\xi_{MM}} + \frac{1}{2} \sum_{i\alpha,j\beta} q_{i\alpha} \frac{dT_{i\alpha,j\beta}}{d\xi_{MM}} q_{j\beta} \tag{S1}$$

where the derivative of the interaction energy $E^{QM/MM}$ is the product between the FQ charges and the electric field, generated by the QM density, that acts on them. The only contribution that arises from the MM energy is due to the derivative of the charge-charge

interaction kernel. In case the Onho kernel[1,2] is employed, the derivative of the kernel can be written as:

$$\frac{dT_{i,j}}{dr_i} = -\frac{\eta_{ij}^3}{(1+\eta_{ij}^2 r_{ij}^2)^{\frac{3}{2}}} r_{ij} \tag{S2}$$

where in this case, i and j are FQ atoms, r_i indicates the position of atom i, $r_{ij} = r_i - r_j$, and η_{ij} is the arithmetic mean between the chemical hardnesses of atom i and atom j.

S2 MD simulations

Molecular dynamics (MD) simulations are carried out for both benzene and phenol in aqueous solution, using the General Amber Force Field (GAFF).[3] GAFF parameters are generated with Antechamber[4] for both solutes, while charges are derived from RESP scheme for benzene[5] and from CM5 model for phenol.[6] Simulations are performed with the GROMACS package.[7] The solvation box is a cube with sides of 5.5 nm, containing about 5300 TIP3P[8] water molecules and one solute molecule in the case of benzene, and about 5600 water molecules in the case of phenol. The steepest descent minimization algorithm is used to minimize energy during the structure's initial relaxation of both solutes. Then, two equilibration steps are carried out. For benzene, an equilibration (0.5 ns) under the NVT ensemble is performed to reach a temperature of 298 K using a coupling constant of 0.1 ps and a velocity-rescaling method,[9] followed by 1 ns of NPT equilibration with the Parrinello–Rahman barostat.[10] For phenol, the corresponding equilibration times are 1 ns (NVT) and 2 ns (NPT). The MD production stage lasts 30 ns in both cases, using the leap-frog integration algorithm[11] with a time step of 1 fs for benzene and 2 fs for phenol. Electrostatic interactions are described with the Particle Mesh Ewald method.[12]

The Radial Distribution Function (RDF) and Dihedral Distribution Function (DDF) for phenol are plotted in Figure S1. The RDF shows that a hydrogen bond is formed between the phenolic hydrogen (H6) and the oxygen atoms of surrounding water molecules, with a pronounced peak centered at 2 Å. The corresponding running coordination number (RCN)

of approximately 1 indicates that, on average, one hydrogen bond is formed. In addition, the C2–C1–O1–H6 dihedral angle, denoted as δ_1, is analyzed along the MD trajectory, showing the expected rotational flexibility of phenolic hydrogen H6, with peaks centered at 0° and ±180°. For comparison, the RDF of benzene is reported in Figure S2, clearly indicating the absence of hydrogen bonding between the benzene hydrogens and the oxygen atoms of water molecules.

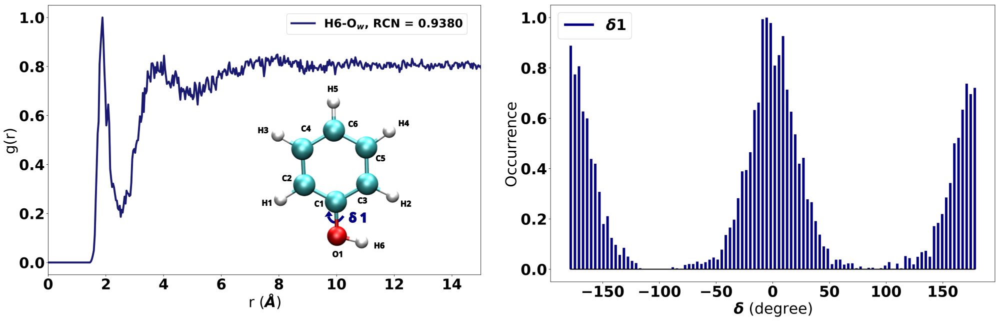

Figure S1: a) Left: Definition of Phenol dihedral angle δ_1, and its corresponding DDF. (b) Right: RDF between the phenol hydrogen (H6) and water oxygen atoms, along with the corresponding running coordination number (RCN).

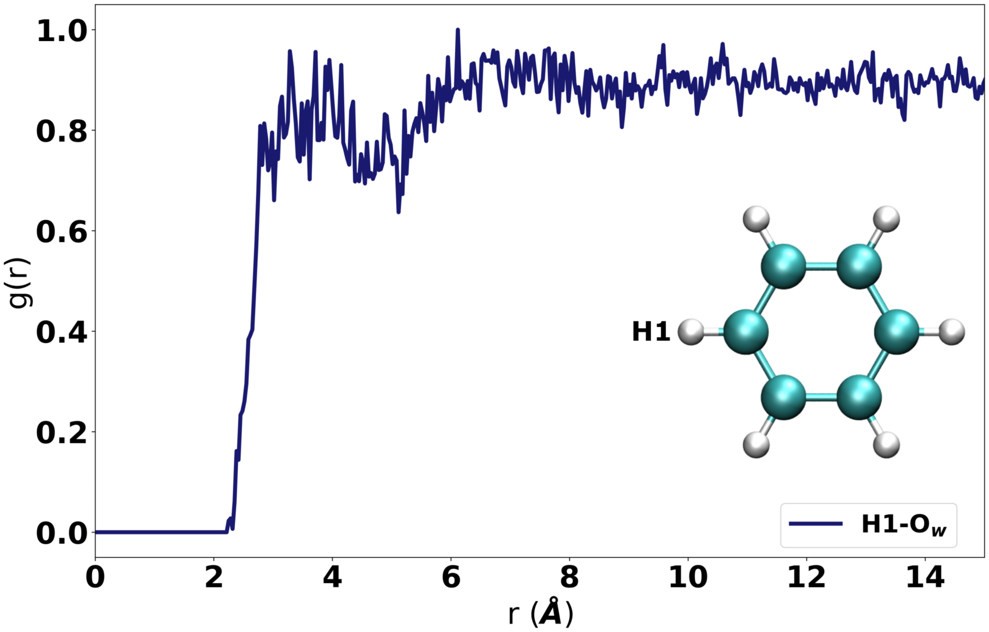

Figure S2: RDF between the benzene hydrogen (H1) and water oxygen atoms.

S3 Active space selection among different frames: MOs overlap

To choose the active space consistently among the different spherical snapshots, a modified version of the protocol presented in Ref. 13 is employed. This procedure aims to select the set of active orbitals manually in a limited number of reference snapshots and then automatically obtain the active space for the rest of the frames (target frames). The idea is to identify the same MOs manually selected in the reference frames in each of the target frames. These orbitals are chosen as the ones that share the maximum overlap with the MOs of the reference frames.

For each target frame, the procedure consists of the following steps:

1. align the structures of the reference and target frames by computing the optimal rotation matrix using the Kabsch algorithm;[14]
2. rotate the MOs accordingly, by using the optimal rotation matrix;
3. compute the overlap matrix;
4. select the MOs with the maximum overlap, in absolute value, with the reference MOs.

According to this procedure, it is only necessary to inspect the active space for frames where the MOs do not exactly match the ones selected in the reference frames (e.g., when the absolute value of the overlap is less than 0.7).

S4 Non-Orthogonality of SS-CASSCF/FQ Wavefunctions

The impact of non-orthogonality on excitation energies can be estimated through Löwdin symmetric orthogonalization, which provides the closest orthogonal set to the original states.[15,16]

Within this method, the vector that contains the orthogonalized wavefunctions $|\boldsymbol{\Psi'}\rangle$ and the vector that contains the original wavefunctions $|\boldsymbol{\Psi}\rangle$ are related by the following equation:

$$|\boldsymbol{\Psi'}\rangle = \boldsymbol{S}^{-1/2}\,|\boldsymbol{\Psi}\rangle \tag{S3}$$

where $\boldsymbol{S}$ is the overlap matrix between the states:

$$\boldsymbol{S} = \begin{pmatrix} 1 & s \\ s & 1 \end{pmatrix} \tag{S4}$$

If $s \ll 1$, $\boldsymbol{S}^{-1/2}$ can be approximated as:

$$\boldsymbol{S}^{-1/2} \approx \begin{pmatrix} 1 & -\frac{s}{2} \\ -\frac{s}{2} & 1 \end{pmatrix} \tag{S5}$$

With this approximation, the orthogonalized states are:

$$\begin{pmatrix} \Psi'_{GS} \\ \Psi'_{ES} \end{pmatrix} = \begin{pmatrix} 1 & -\frac{s}{2} \\ -\frac{s}{2} & 1 \end{pmatrix} \begin{pmatrix} \Psi_{GS} \\ \Psi_{ES} \end{pmatrix} \tag{S6}$$

Considering the orthogonalized states, the new excitation energy of the system is:

$$\begin{aligned} E'_{ES} - E'_{GS} &= \langle \Psi'_{ES}|\hat{H}|\Psi'_{ES}\rangle - \langle \Psi'_{GS}|\hat{H}|\Psi'_{GS}\rangle \\ &\approx E_{ES} - E_{GS} - \frac{s^2}{4}(E_{ES} - E_{GS}) \end{aligned} \tag{S7}$$

where E_{ES} and E_{GS} are the original energies of the ES and GS, respectively. In this equation, an additional approximation has been made, assuming that the GS and ES Hamiltonians are equal. The correction to the excitation energy depends on the square of the overlap term s. Selecting a random snapshot of benzene, the excitation energy of the initial states is $E_{ES} - E_{GS} \approx 0.19$ a.u. and the overlap term is $s = -1.7 \cdot 10^{-3}$ a.u.. This means that

the correction due to the non-orthogonality of the states is within the order of 10^{-7} a.u., therefore it marginally affects the value of the excitation energy. Analogously, considering a random snapshot of phenol in aqueous solution, the excitation energy of the initial states is $E_{ES} - E_{GS} \approx 0.18$ a.u. and the overlap between the two wavefunctions is $s = 1.0 \cdot 10^{-3}$ a.u.. The correction for the excitation energy is therefore of the order of 10^{-8} and also in this case does not substantially affect the value of the excitation energy.

S5 Convergence tests

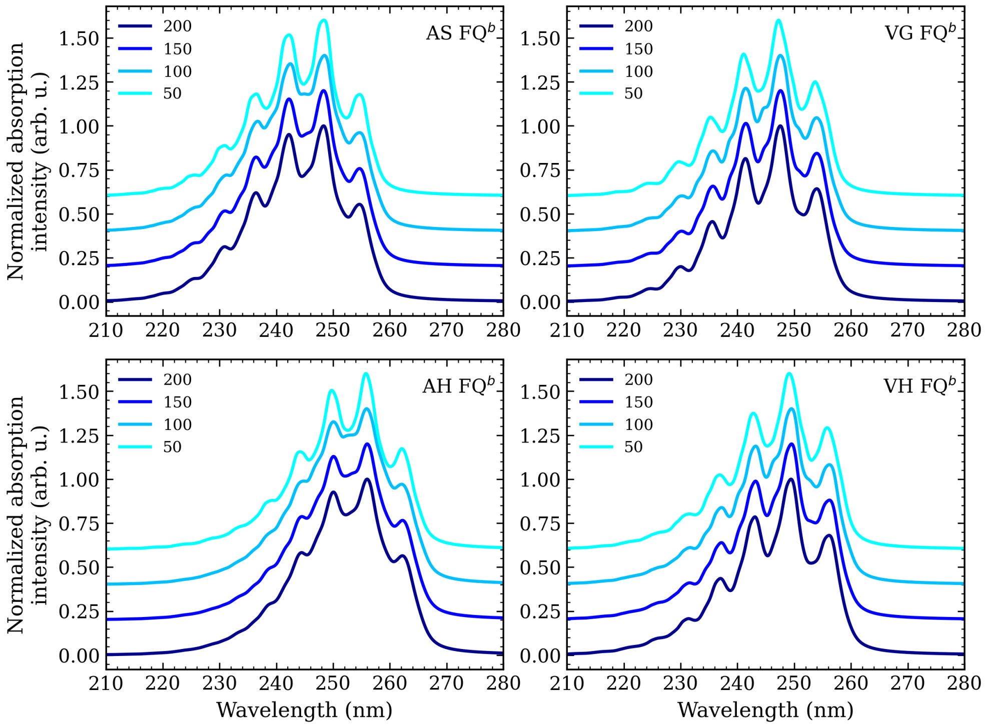

Figure S3: CASSCF(6,6)/FQ^b vibronic spectra of benzene in aqueous solution computed using different harmonic approximations (AS, VG, AH, VH). Each subplot contains 4 stacked spectra obtained by averaging the results of a different number of frames: 50, 100, 150, and 200. Averaged spectra are convoluted with a Lorentzian function with FWHM 0.04 eV.

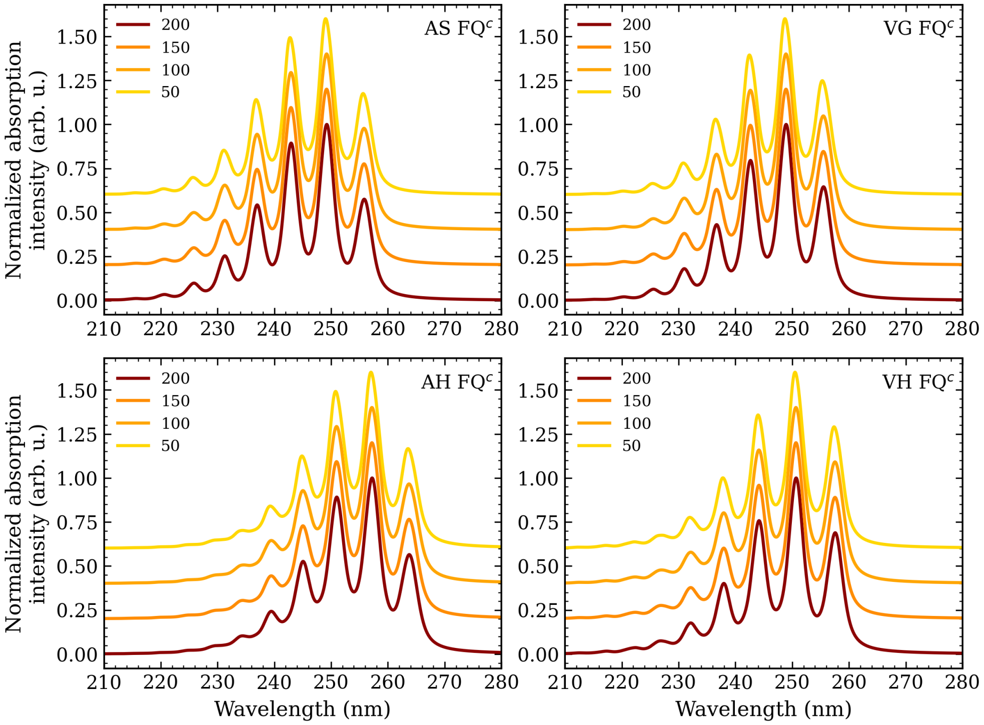

Figure S4: CASSCF(6,6)/FQ^c vibronic spectra of benzene in aqueous solution computed using different harmonic approximations (AS, VG, AH, VH). Each subplot contains 4 stacked spectra obtained by averaging the results of a different number of frames: 50, 100, 150, and 200. Averaged spectra are convoluted with a Lorentzian function with FWHM 0.04 eV.

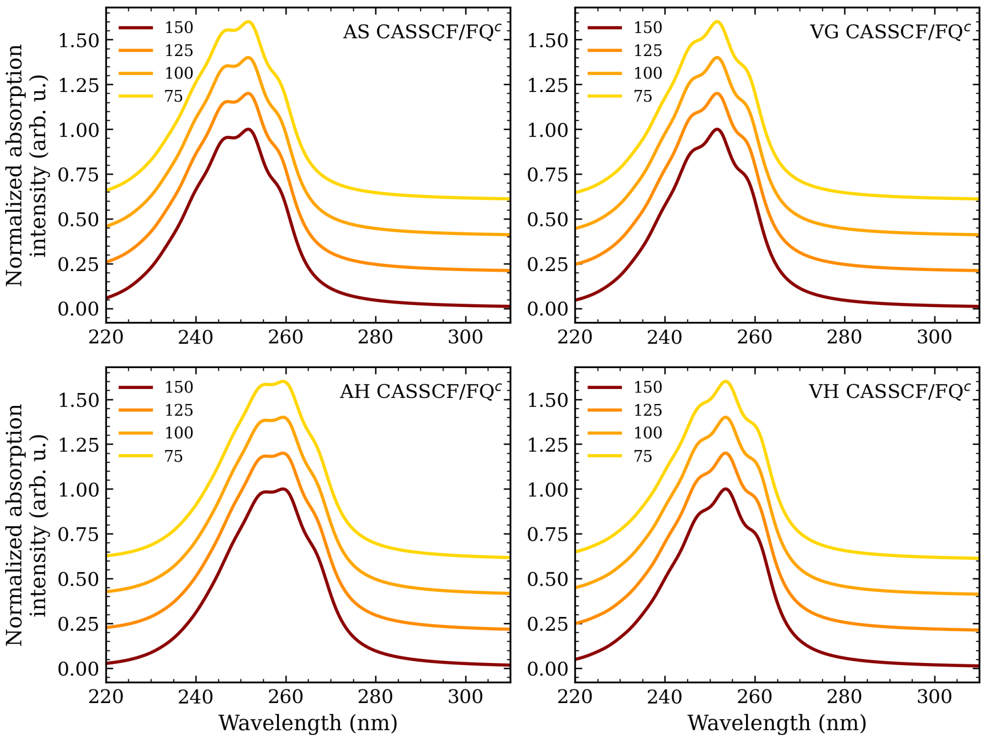

Figure S5: CASSCF(8,7)/FQ^c vibronic spectra of phenol in aqueous solution computed using different harmonic approximations (AS, VG, AH, VH). Each subplot contains 4 stacked spectra obtained by averaging the results of a different number of frames: 75, 100, 125, and 150. Averaged spectra are convoluted with a Lorentzian function with FWHM 0.15 eV.

S6 Vibronic spectra with electrostatic embedding

To further investigate the validity of the newly developed nuclear gradients, some additional calculations using an electrostatic embedding (EE) approach have been performed. These tests are carried out for phenol in aqueous solution, following the same computational protocol presented in Section 3.2 but replacing the FQ polarizable force field with the OPLS-AA[17] force field. The vibronic spectra are computed for 50 snapshots, which are then averaged and convoluted with a Lorentzian function with FWHM of 0.15 eV. The resulting spectra are reported in Figure S6, together with FQ and experimental spectra (the latter is taken from Ref. 18).

EE and FQ stick spectra are in good agreement, both in case of VG and VH calculations. Only a slight difference in the relative intensities between the peaks is observed. This slight difference propagates to the line shape of the convoluted spectrum; in case of EE both the right and the left shoulders are less pronounced with respect to FQ spectra and the left shoulder has a slightly higher relative intensity. Regarding the position of the central peak, EE vibronic spectra are slightly red shifted with respect to FQ, both in case of VG and VH simulations. Overall vibronic spectra obtained with EE or FQ are remarkably similar. These findings suggest that for this specific system the effect of the mutual polarization between the solute and the solvent is less relevant than a proper characterization of the hydrogen bonding pattern that is achieved by resorting to a fully atomistic description of the environment. This similarity can also be attributed to the fact that the FQ^c parameters[19] that are employed in our FQ simulations have been obtained to describe bulk water; as a consequence, the mutual polarization is attenuated with respect to other parametrizations (e.g., FQ^b[20]).

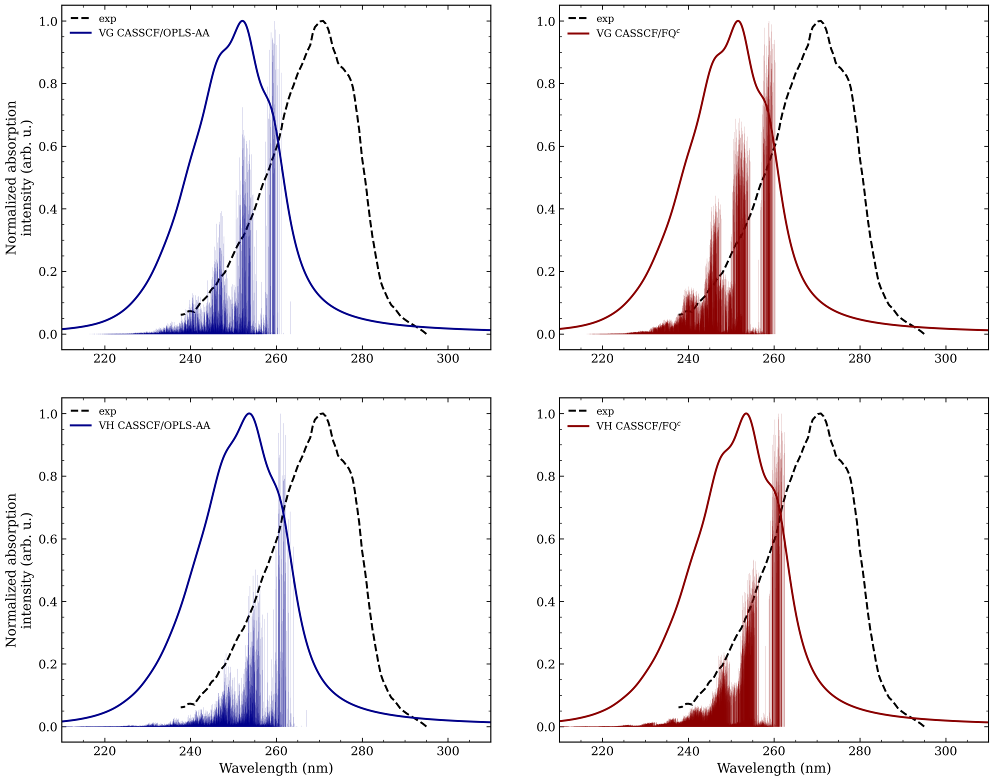

Figure S6: (Left) CASSCF(8,7) vibronic spectra computed by using the EE scheme (OPLS-AA force field), at VG (top) and VH (bottom) level. (Right) CASSCF(8,7) FQ vibronic spectra at VG (top) and VH (bottom) level. Stick spectra are superimposed to spectral profiles. The experimental spectrum adapted from Ref. 18 available under a CC-BY license, Copyright 2018 the Authors, is also shown.

References

(1) Cappelli, C. Integrated QM/polarizable MM/continuum approaches to model chiroptical properties of strongly interacting solute–solvent systems. *Int. J. Quantum Chem.* **2016**, *116*, 1532–1542.

(2) Ohno, K. Some remarks on the Pariser-Parr-Pople method. *Theor. Chem. Acc.* **1964**, *2*, 219–227.

(3) Wang, J.; Wolf, R. M.; Caldwell, J. W.; Kollman, P. A.; Case, D. A. Development and testing of a general amber force field. *J. Comput. Chem.* **2004**, *25*, 1157–1174.

(4) Wang, J.; Wang, W.; Kollman, P. A.; Case, D. A. Automatic atom type and bond type perception in molecular mechanical calculations. *J. Mol. Graph. Model.* **2006**, *25*, 247–260.

(5) Bayly, C. I.; Cieplak, P.; Cornell, W.; Kollman, P. A. A well-behaved electrostatic potential based method using charge restraints for deriving atomic charges: the RESP model. *J. Phys. Chem.* **1993**, *97*, 10269–10280.

(6) Marenich, A. V.; Jerome, S. V.; Cramer, C. J.; Truhlar, D. G. Charge model 5: An extension of Hirshfeld population analysis for the accurate description of molecular interactions in gaseous and condensed phases. *J. Chem. Theory Comput.* **2012**, *8*, 527–541.

(7) Abrahama, M. J.; Murtola, T.; Schulz, R.; Pálla, S.; Smith, J. C.; Hess, B.; Lindahl, E. GROMACS: High Performance Molecular Simulations through Multi-Level Parallelism from Laptops to Supercomputers. *SoftwareX* **2015**, *1-2*, 19–25.

(8) Mark, P.; Nilsson, L. Structure and Dynamics of the TIP3P, SPC, and SPC/E Water Models at 298 K. *J. Phys. Chem. A* **2001**, *105*, 9954–9960.

(9) Bussi, G.; Donadio, D.; Parrinello, M. Canonical sampling through velocity rescaling. *J. Chem. Phys.* **2007**, *126*, 014101.

(10) Parrinello, M.; Rahman, A. Strain fluctuations and elastic constants. *J. Chem. Phys.* **1982**, *76*, 2662–2666.

(11) Ciccotti, G., Hoover, W., Eds. *Molecular - dynamics simulation of statistical - mechanical systems*; Proceedings of the International School of Physics "Enrico Fermi"; North-Holland, 1986.

(12) Darden, T.; York, D.; Pedersen, L. Particle mesh Ewald: An Nlog(N) method for Ewald sums in large systems. *J. Chem. Phys.* **1993**, *98*, 10089–10092.

(13) Cardenas, G.; Nogueira, J. J. An algorithm to correct for the CASSCF active space in multiscale QM/MM calculations based on geometry ensembles. *Int. J. Quantum Chem.* **2021**, *121*, e26533.

(14) Lawrence, J.; Bernal, J.; Witzgall, C. A purely algebraic justification of the Kabsch-Umeyama algorithm. *J. Res. Natl. Inst. Stan.* **2019**, *124*, 1.

(15) Löwdin, P.-O. *Advances in quantum chemistry*; Elsevier, 1970; Vol. 5; pp 185–199.

(16) Aiken, J. G.; Erdos, J. A.; Goldstein, J. A. On löwdin orthogonalization. *Int. J. Quantum Chem.* **1980**, *18*, 1101–1108.

(17) Jorgensen, W. L.; Maxwell, D. S.; Tirado-Rives, J. Development and testing of the OPLS all-atom force field on conformational energetics and properties of organic liquids. *J. Am. Chem. Soc.* **1996**, *118*, 11225–11236.

(18) Riley, J. W.; Wang, B.; Woodhouse, J. L.; Assmann, M.; Worth, G. A.; Fielding, H. H. Unravelling the role of an aqueous environment on the electronic structure and ionization of phenol using photoelectron spectroscopy. *J. Phys. Chem. Lett.* **2018**, *9*, 678–682.

(19) Rick, S. W.; Stuart, S. J.; Berne, B. J. Dynamical fluctuating charge force fields: Application to liquid water. *J. Chem. Phys.* **1994**, *101*, 6141–6156.

(20) Giovannini, T.; Lafiosca, P.; Chandramouli, B.; Barone, V.; Cappelli, C. Effective yet reliable computation of hyperfine coupling constants in solution by a QM/MM approach: Interplay between electrostatics and non-electrostatic effects. *J. Chem. Phys.* **2019**, *150*.
